# Public perceptions of urgency of severe cases of COVID-19 and inflammatory gastrointestinal disease

**DOI:** 10.1371/journal.pone.0273000

**Published:** 2022-08-11

**Authors:** Sarah Koens, Annette Strauß, Jens Klein, Ingmar Schäfer, Olaf von dem Knesebeck

**Affiliations:** 1 Institute of Medical Sociology, University Medical Center Hamburg-Eppendorf, Hamburg, Germany; 2 Department of General Practice and Primary Care, University Medical Center Hamburg-Eppendorf, Hamburg, Germany; University of the Punjab, PAKISTAN

## Abstract

**Background:**

There is evidence that perceived urgency of medical complaints is associated with emergency care utilization. Patients’ perception of urgency can differ from physicians’ assessment. This study explored public perceptions of urgency of severe cases of COVID-19 and inflammatory gastrointestinal disease and analyzed variations in perceptions of urgency by characteristics of the afflicted person in the vignettes and sociodemographic characteristics of respondents.

**Methods:**

Vignettes with severe symptoms of either inflammatory gastrointestinal disease or COVID-19 with comparable urgency of treatment were used in a telephone survey in Germany (N = 1,207). Besides disease, the vignettes varied in terms of sex, age (child, middle-aged person, old person) and daytime (Tuesday morning, Tuesday evening). Respondents were asked to rate the urgency of the reported symptoms with four items. A sum scale was computed. Variations in perceptions of urgency according to vignette characteristics and sociodemographic characteristics of the respondents (sex, age, educational level, migration background, children (yes/no) and personal affliction) were analyzed using a linear regression model.

**Results:**

In terms of vignette characteristics, multivariate analysis showed a lower estimated urgency for males, as well as for the middle-aged and aged persons, compared to the child vignettes, and for COVID-19, compared to inflammatory gastrointestinal disease. Regarding the characteristics of the respondents, estimated urgency increased with age and was lower among respondents, who were previously affected by the symptoms themselves.

**Conclusion:**

Although urgency in the vignettes was comparable, variations in estimated urgency by age and sex of the afflicted person and the described disease as well as age and personal affliction of the respondents were identified. This could result in an inadequate health care service utilization. Therefore, variations in public perceptions of urgency should be considered in the planning of public campaigns on adequate health care services utilization.

## Introduction

Frequent utilization of emergency care or crowding of emergency departments is an issue in various countries [1]. Emergency department overcrowding can lead to reduced patient satisfaction, poorer patient outcomes like increased mortality, delays in assessment and treatment, and stress in nurses and physicians [2,3].

Various studies indicate that many patients visit the emergency department on their own initiative, without prior consultation of a primary care physician and without the advice of a doctor [4–6]. One reason to visit the emergency department directly is the patients’ perception of need [4]. Among others, perceived severity of symptoms determines whether people use medical services, like emergency care [7]. A study reported, that besides anxiety, perceived urgency is a major reason to use emergency care [8]. It was also shown that only in one third of the patients, who used the emergency department, the own perception of urgency matched the physicians’ assessment, with nearly half of the patients perceiving their complaints as more urgent than assessed by triage and 20% perceiving their complaints as less urgent [9].

There are different studies, which analyzed perceived urgency in patients visiting the emergency department because of different medical complaints, but research regarding the general population is rare. Study findings among emergency department patients showed that most of these patients rate their own complaints as urgent [7,10,11]. However, there are inconsistencies, as a German study reported that about half of the patients judged their symptoms as non-urgent [6]. The same study showed that emergency care patients with migration background and older age reported a higher subjective urgency [6]. It was also reported that perceived urgency in emergency care patients may be related to sex, age, education, health status, and previous healthcare experiences [7]. Perceived urgency in emergency care patients seems to be based on the increase and severity of complaints [6], as well as pain intensity [10].

Perception of urgency of symptoms is also related to whether patients can assess what an emergency is [7]. Such knowledge is related to the concept of health literacy that can be defined as “the degree to which individuals have the capacity to obtain, process, and understand basic health information and services needed to make appropriate health decisions” [12]. Health literacy is also associated with the utilization of health services [13]. There is an extensive body of research on the public‘s health literacy in different countries including Germany [14–16], but little is known about public knowledge and beliefs about medical emergencies (‘emergency literacy’).

Apart from the findings on self-perceived urgency in patients visiting emergency departments, to our knowledge there are no studies on perceptions about the urgency of specific severe medical problems in the general population. In light of the problems associated with emergency department crowding and as the perception of urgency is a relevant cause for emergency department visits, it is important to explore public perceptions of urgency and to analyze what factors may influence perceptions of urgency in the general population. With reference to two diseases, COVID-19 and gastrointestinal disease, which are prevalent in the population and common in emergency departments [17–19], we wanted to address the following research questions: (1) How is the urgency of symptoms of severe cases of COVID-19 and inflammatory gastrointestinal disease rated by the public? And (2) does perceived urgency differ by characteristics of the afflicted person and by sociodemographic characteristics of the respondents?

## Methods

### Study design and sample

Analyses are based on cross-sectional data collected via a computer assisted telephone survey between November 2020 and January 2021 in Hamburg, Germany. A random sample was used comprising all possible telephone numbers in Hamburg, including non-registered numbers via random digital dialling [20]. Computer generated, repeated calls were made on different week days by trained interviewers. On the household level, the Kish selection grid [21] was used to randomly choose the target person.

To analyze public perceptions of the urgency of medical cases, 24 vignettes (case stories) were used (please see S1 File). Based on experiences with previous research [22] a number of about n = 50 participants per vignette (i.e. total N = 1,200) was considered sufficient to identify medium-sized differences. Recruitment was continued until the required sample size was reached. 2,756 randomly selected persons (telephone numbers) were included in the net sample. Of these, 961 (34.9%) could not be reached and 588 (21.3%) refused to participate. This led to a total number of 1,207 participants, reflecting a response rate of 43.8%. To compensate for sociodemographic differences, our sample was weighted for sex, age and educational level. Comparisons with official statistics from Hamburg indicated that the weighted sample did not significantly differ from the general adult population regarding the distribution of sex, age, and level of education [23,24] (Table 1).

**Table 1:** Distribution of sociodemographic characteristics of the sample compared with official statistics for the population in Hamburg.

	Sample* (N = 1,207)	Population in Hamburg	P**
Sex (female %)	51.5	51.0	0.625
Age (%) 18–24 Years 25–39 Years 40–59 Years 60–64 Years ≥ 65 Years	9.6 25.9 35.2 7.4 21.9	9.6 28.9 33.5 6.2 21.9	0.113
Education (%) ≤ 9 Years 10 Years ≥ 12 Years	27.1 23.6 49.3	27.0 24.1 48.9	0.924

*weighted.

**p-value of Chi^2^-Tests.

The study was approved by the Local Psychological Ethics Committee at the Center for Psychosocial Medicine, University Medical Center Hamburg (No. LPEK-0200). The respondents were called and informed about the interview and asked for their consent to participate via telephone. If they agreed to participate, the interview was conducted directly afterwards or an appointment for the interview was made. Verbal consent was obtained, because the names and addresses of the participants were not known, only the telephone numbers. Verbal consents and refusals were documented by the interviewers.

### Vignettes

Vignettes were used as a stimulus at the beginning of the survey (please see S1 File). These short case stories were developed in cooperation with primary care physicians, emergency physicians, geriatricians, paediatricians and with nursing staff. Two common disease groups in emergency departments were selected for the vignettes: COVID-19 and inflammatory gastrointestinal diseases. In Germany, abdominal pain is the most common symptom in emergency departments [25]. Acute upper respiratory tract infections are the most frequent complaint in medical on-call services [25]. In contrast to previous years, in 2020 they were among the most common diagnoses in the emergency department in the course of the COVID-19 pandemic [25].

Vignettes were additionally varied according to sex (female, male), age (12 years (child), 49 years (middle-aged person), 72 years (old person)), and daytime (Tuesday, 8 a.m.; Tuesday, 8 p.m.). The resulting 24 vignettes (2x2x3x2) were randomly assigned to the respondents. Presented symptoms of both diseases were severe and comparable regarding urgency of treatment. In terms of inflammatory gastrointestinal symptoms, typical and frequent diseases for the different age groups were selected: appendicitis (child), cholecystitis (middle-aged person), and diverticulitis (old person). In line with the Manchester-Triage-Score, urgency of treatment was indicated by fever or increased temperature and severe pain in all three gastrointestinal vignettes [26]. In case of the COVID-19 symptoms, shortness of breath indicates urgency. Generally, people affected by COVID-19 symptoms should consult a doctor by phone or call the medical on-call service and wait for further instructions, or call the rescue service in case of an emergency [27]. Symptoms of the COVID-19 vignette were based on guidelines [28] and information provided by the Robert Koch Institute [29]. To avoid variations in the presentation of the vignettes, all vignettes were audio-recorded by a person who was trained to speak the texts in a clearly understandable way. These audio files were then directly played to the respondents via the computer.

### Measures

At the beginning of the interview, one of the vignettes was presented to the respondents. To assess perceptions of urgency of treatment in the presented case, four items were developed for this study based on previous studies among patients visiting emergency care facilities [6,8,10]: 1. “The complaints can become life threatening if not treated immediately.” 2. “If such complaints are present, it is an emergency.” 3. “With such complaints, one can first wait and further observe the course.” 4. “These complaints scare me.” On a four-point Likert scale, respondents could indicate their level of agreement or disagreement. With these items, a principal component analysis was carried out (with reverse coding of item 3). All four items loaded on one component with an Eigenvalue of 2.26 (explained variance 56.58%, Cronbach’s α 0.74, mean inter-item correlation 0.42). A sum score ranging from 4–16 was computed with higher scores indicating stronger agreement with urgency perceptions.

The following sociodemographic characteristics of the respondents were introduced as predictors: Age, sex, education (in years of schooling), migration background (yes/no), and having children (yes/no). In terms of education, the respondents were asked about their highest school degree, from which the years of schooling can be derived. As for migration background, the respondents were asked whether they were born in Germany and if their parents were born in Germany. A person has a migration background if the person himself/herself or at least one parent was not born with the German citizenship [30]. Finally, respondents were asked, whether they or their child/children have ever been affected by the complaints which were described in the vignettes (yes/no).

### Analyses

For the urgency perception scale, mean values and standard deviations (sd) are reported. Additionally, median and interquartile ranges (IQR) were computed. Differences in mean scores were tested using analyses of variance (ANOVA) or t-tests. Moreover, the urgency perception scale was entered into a multiple linear regression model as dependent variable. Characteristics of the vignettes (disease, sex, age, and daytime) and of the respondents (sex, age, education, migration status, children, and possible past personal affliction) were entered simultaneously into the models as predictor variables. Estimates and 95% Confidence intervals (CI), standardized coefficients and p-values were calculated. Significant two-way interactions between diseases (COVID-19 vs. inflammatory gastrointestinal diseases) and the other characteristics were also analyzed by interaction-terms, predicted values and pairwise comparisons. The interaction analyses were adjusted for all variables that were entered in the regression model. Conditions for linear regression were checked. The assumptions of linearity and normal distribution of the residuals were met, and no influential observations or collinearity issues for the regression model were identified. The examination of the conditions in the model with interaction terms showed potential collinearity issues, but in case the model includes interaction-terms, high values of the variance inflation factor are expected [31]. The conditions of linearity and normal distribution of the residuals were also met for the model with the interaction terms and no influential observations were identified. Principal component analysis and descriptive analyses were carried out using the Statistical Package for the Social Sciences (SPSS 26) [32]. Multiple linear regression models were performed in the R statistical package [33] including the package emmeans [34], performance [35], and ggplot2 [36]. P-values <0.05 were considered statistically significant. Significant differences are highlighted in bold type.

## Results

### Sample characteristics

Of the 1,207 participants, 51.5% were female, mean age was 48.6 years (sd = 18.76) and 45.9% of the respondents had children. Regarding school education, 27.1% had up to 9 years school education, 23.6% 10 years, and 49.3% at least 12 years. A migration background was reported by 22.7%, and 19.8% of the respondents had been affected by the symptoms reported in the vignettes in the past.

### Perception of urgency by characteristics of the vignettes and the respondents

Table 2 shows the results of the descriptive analysis of the sum scale measuring perceptions of urgency of symptoms. Mean score of urgency was 11.57 (sd: 2.83; range: 12). Estimated urgency was significantly higher for inflammatory gastrointestinal symptoms compared to COVID-19 symptoms (mean: 12.58; sd: 2.59 vs. 10.57; sd: 2.71). For the female vignettes and for the child vignettes, a significantly higher urgency was indicated. In terms of characteristics of the respondents, urgency perception increased with age. Respondents who had children themselves indicated a higher urgency, while people, who had been affected by the described symptoms in the past, indicated a lower urgency. Respondents with 10 years of school education indicated a higher urgency compared to respondents with lower and higher educational level.

**Table 2:** Descriptive analysis of perceptions of urgency of symptoms (sum scale, range 4–16)–results of t-tests and ANOVA^1^^,^^2^.

		Mean (sd)	Median	IQR
	Total (N = 1,135)	11.57 (2.83)	12.00	4.00
**Vignettes**				
Sex	Female (N = 574)	11.76 (2.72)	12.00	4.00
	Male (N = 565)	11.38 (2.93)	12.00	5.00
	p	**0.023**		
Age	Child (N = 384)	12.18 (2.67)	12.21	4.00
	Adult middle aged (N = 399)	10.98 (2.90)	11.00	4.00
	Adult aged (N = 355)	11.58 (2.79)	12.00	4.20
	p	**<0.001**		
Time	Tuesday morning (N = 555)	11.57 (2.87)	12.00	4.00
	Tuesday evening (N = 583)	11.57 (2.80)	12.00	4.00
	p	0.981		
Symptoms	Gastrointestinal (N = 566)	12.58 (2.59)	13.00	4.00
	COVID-19 (N = 572)	10.57 (2.71)	11.00	3.00
	P	**<0.001**		
**Respondents**				
Sex	Male (N = 561)	11.51 (2.8)	12.00	5.00
	Female (N = 577)	11.63 (2.8)	12.00	4.00
	P	0.489		
Age	18–40 (N = 442)	10.97 (2.59)	11.00	4.00
	41–60 (N = 394)	11.76 (2.99)	12.00	4.00
	61 and older (N = 302)	12.20 (2.81)	13.00	4.00
	P	**<0.001**		
Education	≤ 9 years (N = 298)	11.83 (2.72)	12.00	4.00
	10 years (N = 250)	12.02 (2.65)	12.00	4.00
	≥ 12 years (N = 550)	11.25 (2.91)	12.00	4.00
	p	**0.001**		
Migration background	No (N = 869)	11.60 (2.79	12.00	4.00
	Yes (N = 251)	11.48 (2.97)	12.00	5.00
	p	0.549		
Own children	No (N = 611)	11.40 (2.78)	12.00	5.00
	Yes (N = 512)	11.78 (2.90)	12.00	4.00
	p	**0.024**		
Personally affected by such complaints	No (N = 913)	11.78 (2.71)	12.00	4.00
	Yes (N = 223)	10.71 (3.09)	11.00	4.00
	p	**<0.001**		

^1^ weighted.

^2^ p-values <0.05 were considered statistically significant, in bold.

The results of the multiple linear regression are presented in Table 3. Estimated urgency was significantly lower for the male vignettes and for the vignettes with middle-aged and aged persons, compared to the child vignettes, after controlling for all other characteristics of the vignettes and the respondents. For vignettes with inflammatory gastrointestinal symptoms, a higher urgency was indicated compared to the COVID-19 symptoms. Estimated urgency increased with age of the respondents and was significantly lower among respondents, who were previously affected by the symptoms themselves.

**Table 3:** Results of multiple regression analysis of perceptions of urgency of symptoms (N = 1,091)^1^.

Predictors	Estimates	Standardized	95% CI	p^2^
**Vignettes**				
Sex (male)	-0.35	-0.12	-0.66 – -0.05	**0.024**
Age (middleaged)^3^	-1.10	-0.39	-1.47 – -0.73	**<0.001**
Age (aged)^3^	-0.56	-0.20	-0.93 – -0.18	**0.004**
Time (Tuesday evening)	-0.03	-0.01	-0.33 – 0.27	0.833
Symptoms (Covid-19)	-1.97	-0.70	-2.27 – -1.66	**<0.001**
**Respondents**				
Sex (female)	0.10	0.04	-0.20 – 0.41	0.507
Age	0.02	0.14	0.01 – 0.03	**<0.001**
Education (10 years)^4^	0.24	0.09	-0.19 – 0.68	0.272
Education (≥ 12 years)^4^	-0.18	-0.07	-0.56 – 0.19	0.340
Own children (yes)	0.09	0.03	-0.26 – 0.43	0.626
Migration background (yes)	-0.05	-0.02	-0.41 – 0.32	0.806
Affected by such complaints (yes)	-0.62	-0.22	-1.01 – -0.23	**0.002**
**R^2^ / R^2^ adjusted**	0.208 / 0.200			

^1^ weighted.

^2^ p-values <0.05 were considered statistically significant, in bold.

^3^ reference child.

^4^ reference ≤ 9 years.

### Interaction analysis

We identified significant interactions of the symptoms in the vignettes with age of the afflicted person, education of the respondents, respondents’ migration history, and personal affliction. To illustrate these interactions in more detail, predicted values with 95% confidence intervals and Tukey adjusted p-values were computed. The results show that COVID-19 symptoms were consistently rated less urgent than gastrointestinal symptoms (mostly significant differences, please see Table 4). Moreover, COVID-19 symptoms were rated less urgent by respondents who had been affected by the symptoms themselves, while there was no difference in gastrointestinal symptoms. Perceived urgency differed by age in the vignettes in gastrointestinal diseases whereas age in the vignettes had no influence in perception of urgency when COVID-19 symptoms were presented.

**Table 4:** Predicted values (95% CI) of perceptions of urgency of symptoms (N = 1,091)^1^.

	Gastrointestinal	COVID-19	p (Gastrointestinal vs. COVID-19)^2^
**Age (vignettes)**			
Child	13.37 (12.90–13.84)	10.88 (10.46–11.30)	**<0.001**
Adult middle-aged	11.62 (11.20–12.03)	10.59 (10.17–11.00)	**0.043**
Adult aged	12.71 (12.25–13.17)	10.50 (10.08–10.93)	**<0.001**
**Education**			
≤ 9 Years	12.21 (11.75–12.67)	11.11 (10.65–11.57)	0.053
10 Years	12.87 (12.36–13.38)	10.69 (10.21–11.17)	**<0.001**
≥ 12 Years	12.61 (12.20–13.03)	10.17 (9.83–10.52)	**<0.001**
**Migration background**			
No	12.88 (12.55–13.21)	10.30 (10.03–10.57)	**<0.001**
Yes	12.25 (11.76–12.73)	11.02 (10.54–11.50)	**0.006**
**Affected by such complaints**			
No	12.47 (12.21–12.74)	11.22 (10.92–11.52)	**<0.001**
Yes	12.66 (12.07–13.24)	10.10 (9.65–10.54)	**<0.001**

^1^ weighted.

^2^ p-values <0.05 are considered statistically significant, in bold.

## Discussion

### Summary of findings

In this study, we analyzed perceptions of the urgency of COVID-19 symptoms and symptoms of inflammatory gastrointestinal diseases in the general population and investigated differences by characteristics of the respondents and the afflicted person in the vignette. The described symptoms all require timely utilization of medical services to perform diagnostic procedures regardless of gender or age. In terms of all three inflammatory gastrointestinal diseases, hospitalization, either by own initiative or by referral, is required. Although treatment urgency between vignettes was similar, we identified variations in perceptions of urgency regarding characteristics in the vignettes and respondents’ characteristics. In terms of the vignettes, perceived urgency was higher in females and children and when symptoms of inflammatory gastrointestinal diseases were reported. Moreover, perceived urgency increased with the respondents’ age and respondents, who were affected by the described symptoms in the past themselves, considered the reported symptoms less urgent.

### Discussion of results

COVID-19 symptoms were considered less urgent than inflammatory gastrointestinal symptoms. The survey was conducted in winter 2020/2021 during the second wave of the COVID-19 pandemic in Europe [37]. From the beginning of the pandemic, there was a vast amount of information on the disease [38], and the pandemic was discussed intensively [37]. In an online survey on health literacy regarding COVID-19, the majority of participants reported to feel well informed about the disease [38]. Another study showed an association of health literacy with less fear of COVID-19 [39]. However, the sample of the last study cited consisted of medical students and may not be comparable with our sample. Nevertheless, it can be assumed that the population might have been better informed about COVID-19 symptoms compared to symptoms of inflammatory gastrointestinal diseases during the time of the survey. Maybe severe symptoms of COVID-19 are perceived as less urgent compared to severe gastrointestinal symptoms because information about the former is more present in everyday life and because of numerous available information on COVID-19 symptoms. Results of the interaction analysis also showed that COVID-19 symptoms were perceived less urgent in respondents who were previously affected by the complaints themselves. This supports the assumption that symptoms that are known may be perceived as less urgent. Interestingly, analysis of interactions showed that this was not true for inflammatory gastrointestinal symptoms. Generally, since this is the first study analyzing perceptions of urgency in the general population using vignettes, the results are comparable to the existing literature only to a limited extent, as previous studies focussed on patients who visited the emergency department. Our results show an increase in indicated urgency with increasing age of the participants. A study that investigated self-perceived urgency in patients in the emergency department also found a higher self-reported urgency of symptoms in older people [6]. However, in the cited study, the symptoms were not standardized as in this study. Prevalence of multimorbidity in older people is increased, making them vulnerable to various diseases [40,41]. In terms of COVID-19, older people have a higher risk of a severe course of disease [29]. Although the severity of the symptoms in the vignettes was comparable, the overall higher vulnerability might lead to a higher self-perceived urgency in older respondents. In contrast to this, perceived urgency was higher in the child vignettes than in adult persons. Another study reported that parents’ perception of urgency was high when children were affected by illness [42]. Our results show a lower perceived urgency when men are affected, which may indicate that men’s complaints are considered less serious. This and the differences in perceived urgency by age of afflicted people is a problem as it might delay help-seeking and utilization of medical services in men and adult people with severe medical problems.

A study found out, that emergency care utilization was less adequate in people with migration history [43] and it seems likely that perceptions of urgency may play a role to explain this. However, our results do not show differences in urgency perceptions by migration background of respondents. A recent study reported an association between lower health literacy and higher perceived urgency of treatment in emergency department patients [44]. Although an association between higher educational level and higher health literacy or emergency literacy was found [45,46], our findings indicate no differences in perceived urgency by the respondents’ educational level when other characteristics of the respondents as well as of the vignettes were adjusted.

### Limitations

Some methodological aspects have to be considered when interpreting our findings. First, although a response rate of about 44% seems acceptable [47], it has to be considered that more than half of the selected people could not be reached or refused to participate. Therefore, a selection bias cannot be ruled out as non-responders may have different perceptions of urgency of medical complaints. However, we used weighted data that is comparable with official statistics in terms of sex, age and educational level. Second, since this is the first study measuring public perceptions of urgency, there were no validated instruments. A principal component analysis was carried out with four newly developed items. All items loaded on one component and internal consistency (Cronbach’s α 0.74) was acceptable [48]. Third, vignettes are often applied to measure public beliefs and attitudes. They offer the opportunity to give a standardized stimulus because the same symptoms or symptoms with comparable severity are reported. This we consider a strength of the study. However, vignettes have to be short, especially in a telephone survey, as the participants have to remember the case story through the first part of the interview. In order to realistically describe the cases and to present symptoms with comparable severity for two disease groups and three age groups, vignettes were developed in cooperation with clinical experts based on clinical guidelines and official information. Nevertheless, we cannot rule out that participants would have different perceptions of urgency if they or their child really were affected by medically urgent and severe symptoms. In case of inflammatory gastrointestinal disease, the clinical experts recommended to select three different diseases for the three age groups as this was more realistic. However, comparability of the three age groups was limited by this. Finally, the survey was conducted in only one large city in Germany and the results cannot be generalized to rural areas and other countries.

### Conclusions

In previous research on health literacy and emergency care, public perceptions and attitudes about urgency of medical problems have not been taken into account. However, utilization of emergency care seems to be associated with the perception of urgency of medical complaints. Consequently, studies on public perceptions of urgency and predictors of perception of urgency are important. According to our findings, public perceptions about urgency of severe cases seem to depend on sex and age of the afflicted person and the medical condition as well as age and previous personal affliction of respondents. Further research is needed to examine reasons for the identified variations. Additionally, influences of other predictors on perceptions of urgency should be examined. Since perceptions of urgency of symptoms have an impact on utilization of emergency care, differences in perceived urgency could lead to delayed or inadequate use of medical services. Therefore, our findings should be considered in the planning of target group oriented information material and campaigns on adequate utilization of emergency care for the public. For example, campaigns could sensitize for differences in perceptions of urgency by age and gender of afflicted persons, to stimulate the population to reflect possible influences on perceptions of urgency.

## Supporting information

S1 File Case vignettes. (DOCX)

S1 Dataset Data used for analysis. (ZIP)

## References

[1] Pines JM, Hilton JA, Weber EJ, Alkemade AJ, Al Shabanah H, Anderson PD, et al. International perspectives on emergency department crowding. Acad Emerg Med. 2011;18: 1358–1370. doi: 10.1111/j.1553-2712.2011.01235.x 22168200

[2] Morley C, Unwin M, Peterson GM, Stankovich J, Kinsman L. Emergency department crowding: a systematic review of causes, consequences and solutions. Bellolio F, editor. PLoS ONE. 2018;13: e0203316. doi: 10.1371/journal.pone.0203316 30161242 PMC6117060

[3] Carter EJ, Pouch SM, Larson EL. The relationship between emergency department crowding and patient outcomes: a systematic review. J Nurs Scholarsh. 2014;46: 106–115. doi: 10.1111/jnu.12055 24354886 PMC4033834

[4] Afilalo J. Nonurgent emergency department patient characteristics and barriers to primary care. Acad EmergMed. 2004;11: 1302–1310. doi: 10.1197/j.aem.2004.08.032 15576521

[5] van der Linden MC, Lindeboom R, van der Linden N, van den Brand CL, Lam RC, Lucas C, et al. Self-referring patients at the emergency department: appropriateness of ED use and motives for self-referral. Int J Emerg Med. 2014;7: 28. doi: 10.1186/s12245-014-0028-1 25097670 PMC4110705

[6] Scherer M, Lühmann D, Kazek A, Hansen H, Schäfer I. Patients attending emergency departments. Dtsch Arztebl Int. 2017;114: 645–652. doi: 10.3238/arztebl.2017.0645 29034865 PMC5651827

[7] Uscher-Pines L, Pines J, Kellermann A, Gillen E, Mehrotra A. Emergency department visits for nonurgent conditions: systematic literature review. Am J Manag Care. 2013;19: 47–59. 23379744 PMC4156292

[8] Coster JE, Turner JK, Bradbury D, Cantrell A. Why do people choose emergency and urgent care services? A rapid review utilizing a systematic literature search and narrative synthesis. Acad Emerg Med. 2017;24: 1137–1149. doi: 10.1111/acem.13220 28493626 PMC5599959

[9] Toloo G-S, Aitken P, Crilly J, FitzGerald G. Agreement between triage category and patient’s perception of priority in emergency departments. Scand J Trauma Resusc Emerg Med. 2016;24: 126. doi: 10.1186/s13049-016-0316-2 27756416 PMC5070359

[10] Somasundaram R, Geissler A, Leidel B, Wrede C. [Motivations for emergency department use—results of a patient survey]. Gesundheitswesen. 2018;80: 621–627. doi: 10.1055/s-0042-112459 27611882

[11] Berkman ND, Sheridan SL, Donahue KE, Halpern DJ, Crotty K. Low health literacy and health outcomes: an updated systematic review. Ann Intern Med. 2011;155: 97. doi: 10.7326/0003-4819-155-2-201107190-00005 21768583

[12] Selden C, Zorn M, Ratzan R, Parker R. Health literacy [bibliography online]. In: National Library of Medicine [Internet]. 2000 [cited 14 Jun 2021]. Available: https://www.nlm.nih.gov/archive/20061214/pubs/cbm/hliteracy.html.

[13] Sørensen K, Van den Broucke S, Fullam J, Doyle G, Pelikan J, Slonska Z, et al. Health literacy and public health: a systematic review and integration of definitions and models. BMC Public Health. 2012;12: 80. doi: 10.1186/1471-2458-12-80 22276600 PMC3292515

[14] Sørensen K, Pelikan JM, Röthlin F, Ganahl K, Slonska Z, Doyle G, et al. Health literacy in Europe: comparative results of the European health literacy survey (HLS-EU). Eur J Public Health. 2015;25: 1053–1058. doi: 10.1093/eurpub/ckv043 25843827 PMC4668324

[15] Kutner M, Greenberg E, Jin Y, Paulsen C. The health literacy of America’s adults: results from the 2003 national assessment of adult literacy. Washington D.C.: Department of Education, National Centre for Education Statistics; 2006. Report No.: Report N.: NCES 2006–483.

[16] Schaeffer D, Berens E-M, Vogt D. Health literacy in the German population. Dtsch Arztebl Int. 2017;114: 53–60. doi: 10.3238/arztebl.2017.0053 28211318 PMC5319379

[17] Peery AF, Crockett SD, Barritt AS, Dellon ES, Eluri S, Gangarosa LM, et al. Burden of gastrointestinal, liver, and pancreatic diseases in the United States. Gastroenterology. 2015;149: 1731–1741.e3. doi: 10.1053/j.gastro.2015.08.045 26327134 PMC4663148

[18] World Health Organization. WHO coronavirus (COVID-19) Dashboard. 2022 [cited 29 Mar 2022]. Available: https://covid19.who.int/.

[19] Myer PA, Mannalithara A, Singh G, Singh G, Pasricha PJ, Ladabaum U. Clinical and economic burden of emergency department visits due to gastrointestinal diseases in the United States. Am J Gastroenterol. 2013;108: 1496–1507. doi: 10.1038/ajg.2013.199 23857475

[20] Häder S, Gabler S. [A new sample design for telephone surveys in Germany]. In: Gabler S, Häder S, Hoffmeyer-Zlotnik JHP, editors. [Telephone sampling in Germany]. Wiesbaden: VS Verlag für Sozialwissenschaften; 1998. pp. 69–88.

[21] Kish L. A procedure for objective respondent selection within the household. Journal of the American Statistical Association. 1949;44: 380–387. doi: 10.1080/01621459.1949.10483314

[22] von dem Knesebeck O, Löwe B, Lehmann M, Makowski AC. Public beliefs about somatic symptom disorders. Front Psychiatry. 2018;9: 616. doi: 10.3389/fpsyt.2018.00616 30515112 PMC6255972

[23] Statistical Office for Hamburg and Schleswig-Holstein. Hamburg statistical yearbook 2019/2020. Hamburg: Statistical Office for Hamburg and Schleswig-Holstein; 2020.

[24] Federal Statistical Office. Education level of the population—results of the microcensus 2019 -. Wiesbaden: Federal Statistical Office; 2020.

[25] Mangiapane S, Czihal T, von Stillfried D. [Development of outpatient emergency care in Germany from 2009 to 2020]. Berlin: [Central Institute for the Provision of Health Care by Statutory Health Insurance Physicians in the Federal Republic of Germany]; 2021 [cited 1 Jun 2021]. Available: https://www.zi.de/fileadmin/images/content/Publikationen/Zi-Paper-16-2021-Notfallversorgung.pdf.

[26] Schellein O, Ludwig-Pistor F, Bremerich DH. ["Manchester Triage System": process optimization in the interdisciplinary emergency department]. Anaesthesist. 2009;58: 163–170. doi: 10.1007/s00101-008-1477-9 19082988

[27] [Federal Ministy of Health]. [Together against Corona]. 2022 [cited 24 Mar 2022]. Available: https://www.zusammengegencorona.de/informieren/basiswissen-zum-coronavirus/ich-habe-corona-was-soll-ich-tun/.

[28] Blankenfeld H, Kaduszkiewicz H, Kochen MM, Pömsl J. [New coronavirus (SARS-CoV-2) -Information for family practice. DEGAM S1-recommendation for action]. Version 18. Ulm: [German Society for General and Family Medicine e.V.]; 2021 [cited 1 Jun 2021]. Available: https://www.awmf.org/uploads/tx_szleitlinien/054-054l_S1_Neues_CORONA_Virus_2021-05.pdf.

[29] Robert Koch-Institut. [Epidemiological profile of SARS-CoV-2 and COVID-19]. 19 Apr 2021 [cited 10 May 2021]. Available: https://www.rki.de/DE/Content/InfAZ/N/Neuartiges_Coronavirus/Steckbrief.html;jsessionid=3930C7C79979318992F6E9A2704C2C47.internet122?nn=13490888#doc13776792bodyText8.

[30] Federal Statistical Office. [Migration and integration—migration background]. 2021 [cited 20 Sep 2021]. Available: https://www.destatis.de/DE/Themen/Gesellschaft-Umwelt/Bevoelkerung/Migration-Integration/Glossar/migrationshintergrund.html.

[31] Francoeur RB. Could sequential residual centering resolve low sensitivity in moderated regression? Simulations and cancer symptom clusters. OJS. 2013;03: 24–44. doi: 10.4236/ojs.2013.36A004

[32] Corp IBM. IBM SPSS Statistics for Windows, Version 26.0. Armonk, NY: IBM Corp.; 2019.

[33] R Core Team. R: a language and environment for statistical computing. Vienna: R foundation for statistical computing; 2021. Available: https://www.r-project.org/.

[34] Lenth R, Buerkner P, Herve M, Love P, Riebl H, Singmann H. emmeans: estimated marginal means, aka least-squares means. 2021. Available: https://github.com/rvlenth/emmeans.

[35] Lüdecke D, Ben-Shachar M, Patil I, Waggoner P, Makowski D. performance: an R package for assessment, comparison and testing of statistical models. JOSS. 2021;6: 3139. doi: 10.21105/joss.03139

[36] Wickham H. ggplot2: elegant graphics for data analysis. 2nd ed. 2016. Dodrecht, Heidelberg, London, New York: Springer; 2016. doi: 10.1007/978-3-319-24277-4

[37] Graichen H. What is the difference between the first and the second/third wave of Covid-19?–German perspective. J Orthop. 2021;24: A1–A3. doi: 10.1016/j.jor.2021.01.011 33519131 PMC7838578

[38] Okan O, Bollweg TM, Berens E-M, Hurrelmann K, Bauer U, Schaeffer D. Coronavirus-related health literacy: a cross-sectional study in adults during the COVID-19 infodemic in Germany. IJERPH. 2020;17: 5503. doi: 10.3390/ijerph17155503 32751484 PMC7432052

[39] Nguyen HT, Do BN, Pham KM, Kim GB, Dam HTB, Nguyen TT, et al. Fear of COVID-19 scale—associations of its scores with health literacy and health-related behaviors among medical students. IJERPH. 2020;17: 4164. doi: 10.3390/ijerph17114164 32545240 PMC7311979

[40] Rizza A, Kaplan V, Senn O, Rosemann T, Bhend H, Tandjung R. Age- and gender-related prevalence of multimorbidity in primary care: the swiss fire project. BMC Fam Pract. 2012;13: 113. doi: 10.1186/1471-2296-13-113 23181753 PMC3557138

[41] Salive ME. Multimorbidity in older adults. Epidemiol Rev. 2013;35: 75–83. doi: 10.1093/epirev/mxs009 23372025

[42] Kalidindi S, Mahajan P, Thomas R, Sethuraman U. Parental perception of urgency of illness: Pediatr Emerg Care. 2010;26: 549–553. doi: 10.1097/PEC.0b013e3181ea71b3 20657341

[43] Sauzet O, David M, Naghavi B, Borde T, Sehouli J, Razum O. Adequate utilization of emergency services in Germany: is there a differential by migration background? Front Public Health. 2021;8: 613250. doi: 10.3389/fpubh.2020.613250 33490025 PMC7820806

[44] Wehler M, Kalch A, Bilandzic H, Händl T. [Health literacy and nonurgent emergency department visitors]. Notfall Rettungsmed. 2021. doi: 10.1007/s10049-021-00859-z 33786013 PMC7993414

[45] van der Heide I, Wang J, Droomers M, Spreeuwenberg P, Rademakers J, Uiters E. The relationship between health, education, and health literacy: results from the Dutch adult literacy and life skills survey. J Health Commun. 2013;18: 172–184. doi: 10.1080/10810730.2013.825668 24093354 PMC3814618

[46] von dem Knesebeck O, Koens S, Schäfer I, Strauß A, Klein J. Public knowledge about emergency care—results of a population survey from Germany. Front Public Health. 2022;9: 787921. doi: 10.3389/fpubh.2021.787921 35071168 PMC8777036

[47] Curtin R, Presser S, Singer E. Changes in telephone survey nonresponse over the past quarter century. Public Opinion Quarterly. 2005;69: 87–98. doi: 10.1093/poq/nfi002

[48] Tavakol M, Dennick R. Making sense of Cronbach’s alpha. Int J Medical Education. 2011;2: 53–55. doi: 10.5116/ijme.4dfb.8dfd 28029643 PMC4205511

