# Supplementary material for: Public perceptions of urgency of severe cases of COVID-19 and inflammatory gastrointestinal disease
Source: PLoS One. 2022 Aug 11;17(8):e0273000. doi: 10.1371/journal.pone.0273000 (PMC9371268; doi:10.1371/journal.pone.0273000)
Supplement: S1 File — (DOCX) [file pone.0273000.s001.docx]

**Supplement 1:** Case vignettes

| **Symptoms: COVID-19** |
| --- |
| **Age group: children** |
| *It is Tuesday morning, 8 a.m./It is Tuesday evening, 8 p.m., Paula S./Lukas P., 12 years old, schoolgirl/ schoolboy,*  …has had a headache for three days. She has developed a dry cough since yesterday. She feels tired and lacks the drive to do something which is unusual for the girl). She did not go to school today. Her mother took her temperature, it is 38.7 °C. Additionally, since today, Paula has trouble breathing when climbing stairs. Her parents are very worried because this has never happened before. |
| **Age group: adults, middle-aged** |
| *It is Tuesday morning, 8 a.m./It is Tuesday evening, 8 p.m., Melanie P./Stefan D., 49 years, administration officer,*  …has had a headache for three days. She has developed a dry cough since yesterday. She feels tired and lacks the drive to do something which is unusual for her. She did not go to work today. Mrs. P.‘s temperature is at 38.7 °C today. Additionally, since today, she has trouble breathing when climbing stairs. She is very worried because this has never happened before. |
| **Age group: adults, elder** |
| *It is Tuesday morning, 8 a.m./It is Tuesday evening, 8 p.m., Hildegard S./ Helmut K., 72 years, retired,* …has had a headache for three days. She has developed a dry cough since yesterday. She feels tired and lacks the drive to do something which is unusual for her. Mrs. S’s temperature is at 38.7 °C today. Additionally, since today, she has trouble breathing when climbing stairs. Seeing that her heart and lungs are actually in good condition, this worries her very much. |

| **Symptoms: Inflammatory gastrointestinal diseases** |
| --- |
| **Age group: children (appendicitis)** |
| *It is Tuesday morning, 8 a.m./It is Tuesday evening, 8 p.m., Paula S./Lukas P., 12 years old, schoolgirl/ schoolboy,*  …complains about a bad tummy ache. The trouble started the evening before with sickness and pain at the center of the stomach. In the morning she had to vomit. Since the evening before the pain has become worse and has moved to the right lower side of the stomach. The otherwise so active girl feels weak, has no appetite and would rather not move at all. Her temperature was 37.4 °C. When Paula’s parents tried to palpate her tummy, she screamed of pain. This worries her parents very much. |
| **Age group: adults, middle-aged (cholecystitis)** |
| *It is Tuesday morning, 8 a.m./It is Tuesday evening, 8 p.m., Melanie P./Stefan D., 49 years, administration officer,*  …has sudden, severe pain in his right upper abdomen which radiates right up to his back and the right shoulder. Besides, she feels sick but so far did not have to vomit. Because of her bad condition she took her temperature: 38.6 °C. Her pain started about two hours ago. Mrs. P. does not know (has not experienced) pain like this. This worries her very much. |
| **Age group: adults, elder (diverticulitis)** |
| *It is Tuesday morning, 8 a.m./ It is Tuesday evening, 8 p.m., Hildegard S./ Helmut K., 72 years, retired,*  *…has had pain in the lower left abdomen for three days.* For a few weeks she has occasionally suffered from diarrhea, constipation also occurs. She feels less productive than usual and is often tired. Because of her bad condition she took her temperature: 38.5 °C. She noticed a streak of fresh blood on her stool since yesterday. Today the pain has reached a strength she had not experienced before. This very much worries her. |
